# What Have We Learned from Design of Function in Large Proteins?

**DOI:** 10.34133/2022/9787581

**Published:** 2022-03-08

**Authors:** Olga Khersonsky, Sarel J. Fleishman

**Affiliations:** Department of Biomolecular Sciences, Weizmann Institute of Science, Rehovot 7610001, Israel

## Abstract

The overarching goal of computational protein design is to gain complete control over protein structure and function. The majority of sophisticated binders and enzymes, however, are large and exhibit diverse and complex folds that defy atomistic design calculations. Encouragingly, recent strategies that combine evolutionary constraints from natural homologs with atomistic calculations have significantly improved design accuracy. In these approaches, evolutionary constraints mitigate the risk from misfolding and aggregation, focusing atomistic design calculations on a small but highly enriched sequence subspace. Such methods have dramatically optimized diverse proteins, including vaccine immunogens, enzymes for sustainable chemistry, and proteins with therapeutic potential. The new generation of deep learning-based ab initio structure predictors can be combined with these methods to extend the scope of protein design, in principle, to any natural protein of known sequence. We envision that protein engineering will come to rely on completely computational methods to efficiently discover and optimize biomolecular activities.

## 1. Introduction

The high versatility and specificity that protein binders and enzymes exhibit make them exceptionally attractive in biomolecular research, medicine, and biotechnology. Fields as diverse as biomedical engineering [1], sustainable chemistry [2], and commodity production [3] have come to rely on proteins to provide efficient, economical, and environmentally sustainable solutions. These fields are likely to increasingly focus on proteins due to the pressing need to minimize energy use and the environmental impact of industrial processes. As a rule, however, natural proteins very rarely meet the stringencies of real-world applications. For instance, depending on the source organism, as many as half of nonmembrane proteins exhibit low solubility in heterologous expression systems [4, 5], limiting their usefulness even in basic research let alone in applications. Furthermore, with the exception of proteins from thermophilic organisms, many proteins are not stable at high temperatures or in long-term storage and may exhibit suboptimal levels of activity or selectivity [6]. Thus, natural proteins present enormous opportunities to control the “chemistry of life”—the intricate molecular processes that underlie life processes—but a host of obstacles related to stability and activity must be overcome to study and exploit them. Understanding the underlying biophysical reasons for these obstacles and developing a general strategy to address them have been the subjects of immense basic and applied interest for decades [7–9].

Atomistic protein design strategies are based on the thermodynamic hypothesis [10], which stipulates that the protein native-state energy must be lower than that of any competing misfolded or unfolded states. Accordingly, design calculations search for a sequence and conformation that exhibit low native-state energy [11]. Early successes in the atomistic design of stable and accurate protein structures [12], including of a completely new fold [13] led to optimism that protein design would replace traditional, iterative, and laborious protein optimization methods with a completely rational approach. According to this view, if stable new-to-nature folds could be designed at atomic accuracy, it stood to reason that general and reliable atomistic methods for stabilizing proteins or altering their activities could not be far from reach.

Nevertheless, atomistic design of natural proteins exhibited only limited success. Binder and enzyme designs almost always exhibited only a low level of activity and demanded intensive iterative experimental optimization to reach acceptable levels [14–19]. Furthermore, experimental structures often revealed significant differences from the design conception, including substantial and unpredicted deviations in backbone conformation or active-site sidechain constellations [20–22]. These inaccuracies highlighted a fundamental challenge to protein design methodology; namely, a prerequisite to the successful design of function is accurate control over all conformational degrees of freedom. Phrased more precisely, the key determining question for protein design methodology has been to identify a general computational strategy to encode the significant energy gap between native and nonnative protein conformations [8, 11, 23].

The number of possible nonnative conformations scales exponentially with the size of the protein [24]. Thus, small proteins may not exhibit many nonnative alternative states that must be countered in design, and such proteins are therefore more amenable to complete computational design [11]. Indeed, over the past decade, impressive progress was made in understanding the folding and stability of small *de novo* designed proteins (typically <90 amino acids) or idealized versions of natural folds [11, 25–28]. Such proteins can now be generated completely on the computer, though they exhibit no significant sequence relationship to natural proteins [29]. These successes demonstrate significant progress and a high level of understanding and control over the fundamentals of protein folding. We refer the reader to excellent recent reviews on *de novo* designed proteins [28, 30–33].

Despite these dramatic achievements, however, reliable and fully atomistic design of large proteins of a complex fold has not made comparable breakthroughs. Certainly, protein engineering studies often use computational design calculations to focus experiments or to construct “smart” libraries for experimental screening [34–39]. Due to the limited accuracy of the atomistic design calculations, however, these workflows typically iterate computations with experimental screening and structure determination and do not provide a complete computational optimization solution. Critically, they also demand system-specific expertise and are difficult to generalize to proteins for which such expertise has not yet been developed. Thus, the key question that has guided our research is whether there may be general design principles that can be universally applied to proteins of all folds and sizes?

## 2. Design Essentials in Large Proteins

**Figure 1:**
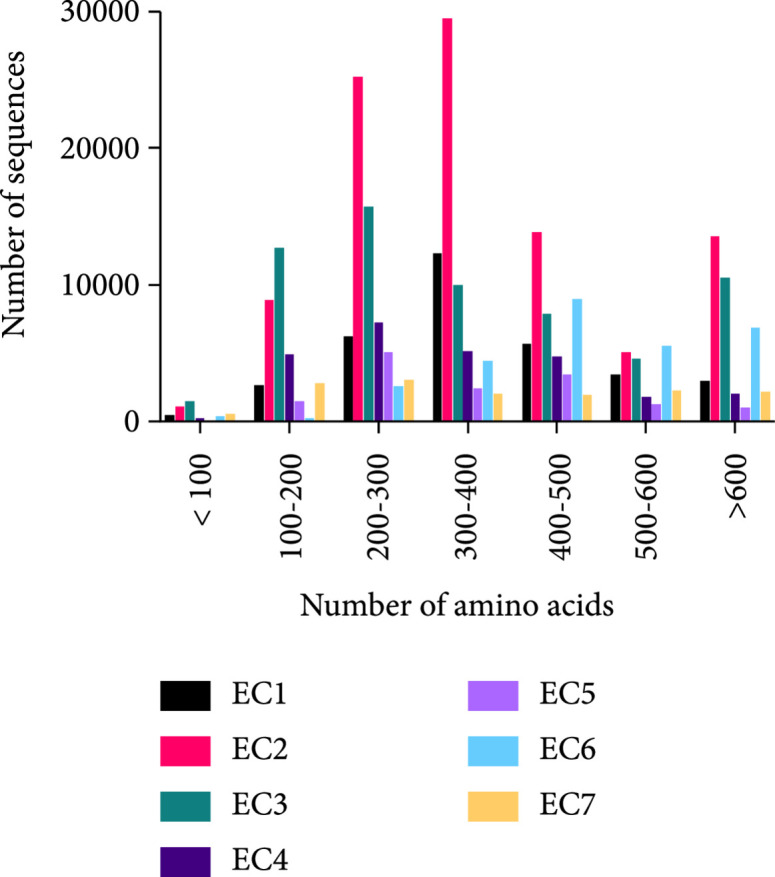
Natural proteins tend to be large. Enzymes in all Enzyme Classifications (EC) exhibit a mean size of more than 200 amino acids. Data downloaded from the UniProt database (https://www.uniprot.org/). Proteins with<100 amino acids (<2% of the entries) may include subdomain fragments, and those with>600 amino acids may include multichain complexes.

Natural proteins tend to be large. The average size of proteins in all organisms is approximately 250 amino acids (350 in eukaryotes), and fewer than 2% of natural proteins are smaller than 100 amino acids [40]. Furthermore, regardless of their functional class, enzymes tend to have large sizes (Figure 1). A possible explanation for this propensity to large size is that proteins evolve through the accretion of subdomain fragments [41, 42]—an inefficient process of exploring the space of potential folds that may result in structural redundancy. Although this argument is plausible, we favor the view that proteins are large for a fundamental reason: many enzymes and binders must encode destabilizing molecular features in their active sites, such as desolvated nucleophilic or acidic amino acids [6, 43]. Accurately positioning such features demands a significant thermodynamic compensation from large regions outside the protein active site. Thus, even though an active site may comprise only a handful of amino acids, its accurate formation may require hundreds of amino acids that fold into a low-energy state. Other important functional features, such as regulatory sites, and large ligand interaction surfaces may also demand large protein sizes.

**Figure 2:**
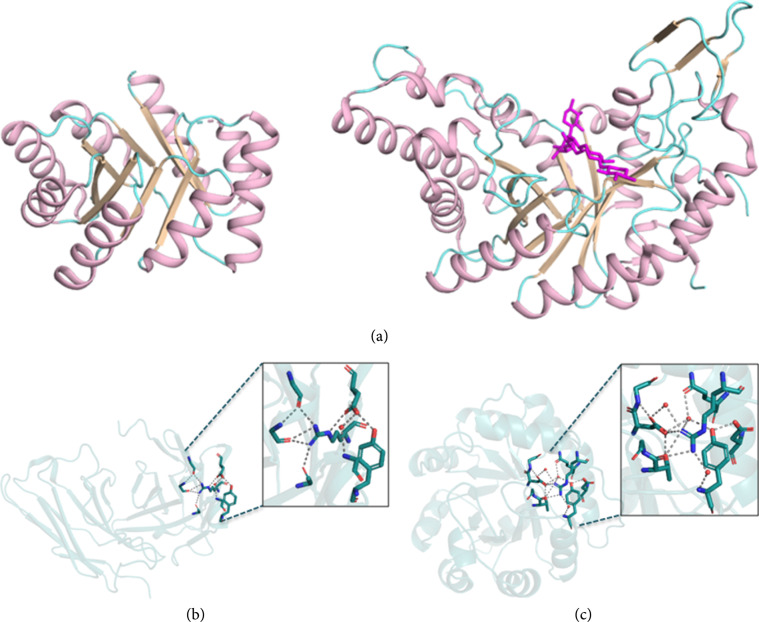
Natural proteins comprise conserved but nonideal features. (a) (Left) A *de novo* designed ideal TIM barrel fold protein (PDB entry: 5bvl; 184 amino acids) [38] *vs.* (right) a natural TIM barrel fold glycoside hydrolase (PDB entry: 4pud; 379 amino acids). Natural TIM barrels comprise large loop structures in their active sites whereas *de novo* designs comprise mostly secondary-structure elements. Structures are colored according to secondary-structure elements with helices, *β* sheets, and loops in pink, wheat, and cyan, respectively. Dashed lines indicate regions that lack electron density. The glycoside hydrolase substrate is indicated in magenta sticks. The two structures are drawn to scale. (b, c) Natural proteins often exhibit intricate buried hydrogen bond networks that may specify their folds. (b) Structure of the variable domain of antibody 4m5.3 (PDB entry: 1x9q) shows a network of buried polar and charged amino acids mediating contacts between regions that are distant on the primary sequence within the heavy chain. (c) A buried polar network in an indole glycerol phosphate synthase (PDB entry: 1a53). Putative polar bonds are shown as dashed lines, and water molecules are indicated by red spheres.

As we were studying how to design large proteins of a complex fold, we hoped to find molecular features that were common to diverse proteins [44]. But one of the obstacles to inferring general protein design principles is the sheer diversity of protein structures. Protein domains are classified by SCOP into more than 1,500 folds and 2,500 superfamilies [45]. Nevertheless, we were struck by the fact that the proteins we studied invariably broke some of the fundamental rules that have been successfully implemented in *de novo* design methodology. First, many protein folds, particularly the functionally more versatile ones like TIM barrels, *β* propellers, and immunoglobulins, comprise long loops at the active sites. By contrast, *de novo* designed proteins are typically dominated by secondary-structure elements that are connected through very short unstructured linkers [13, 29, 46] (Figure 2(a)). Critically, in natural proteins, irregular but structured backbone loops often form large parts of the active site and have important functional roles. Second, in *de novo* design methodology, amino acid positions in the protein core are programmed to exhibit only hydrophobic identities [25, 29] bolstering the hydrophobic effect which is one of the primary driving forces for protein folding [6]. Nevertheless, in every natural fold we examined, we found polar and even charged amino acid residues buried in the protein core [44, 47–49] (Figures 2(b) and 2(c)). The buried polar amino acids often interact with the loop backbones (Figure 2(b)), suggesting that these amino acids are important for the structural stability of the irregular loop regions. We also found that these buried polar amino acids are evolutionarily conserved among homologs, further suggesting that they have an essential structural role [50]. Since hydrogen bond networks demand high structural precision and often link distant parts of the protein sequence, we were intrigued that they might provide a powerful mechanism to specify the backbone conformation in large proteins of a complex fold. They could thereby accentuate the energy gap between the native and nonnative states that is the hallmark of natural proteins [11]. Thus, our working hypothesis was that these buried polar networks, though they diverge from ideality, may hold a key to the problem of designing large and complex folds.

## 3. Backbone Design in Large and Functional Proteins

To test this hypothesis, we developed a general strategy for protein backbone design in large proteins through the assembly of subdomain fragments [48, 51]. In this work, we were inspired by bioinformatics studies that had demonstrated that large natural folds likely emerged by the accretion of small subdomains [41, 42, 52, 53]. Furthermore, protein engineering studies had implemented this strategy in the lab, demonstrating that fragments could be recombined to generate new proteins with different stability and specificity profiles [54–59]. Recently, assembly strategies have also been applied to extend the size of *de novo* designed proteins [60–64]. Nevertheless, recombination events can lead to structural inaccuracy in the form of “hopeful monsters” [65] and typically yield proteins that exhibit low (or no) activity. Thus, accuracy and control over the outcome of the assembly and design process are significant challenges.

In our work, we asked whether we could design new backbones and functional proteins through an evolution-guided atomistic design approach [48, 49, 51]. In this approach, following the assembly of backbone fragments, we subjected the entire protein to atomistic sequence design. Here, we were conscious that atomistic design calculations were very likely to eliminate crucial hydrogen bonding networks in the protein core. Therefore, instead of allowing all amino acid choices at each position during the sequence design phase, we biased design calculations to mutations commonly observed among homologs and forbade rare mutations.

As a first test, we applied this strategy to the design of antibody variable domains. Antibodies were recognized as modular proteins already in the 1970s [66], leading to a wave of innovation in therapeutic antibody engineering [67]. We demonstrated that antibody variable domains designed strictly according to Rosetta atomistic design calculations eliminated conserved and critical buried hydrogen bond networks and exhibited very low protein expression levels [49]. By contrast, by applying evolutionary sequence constraints, we retained these critical networks, generating highly expressed and structurally accurate antibodies that exhibited dozens of mutations from any natural antibody.

**Figure 3:**
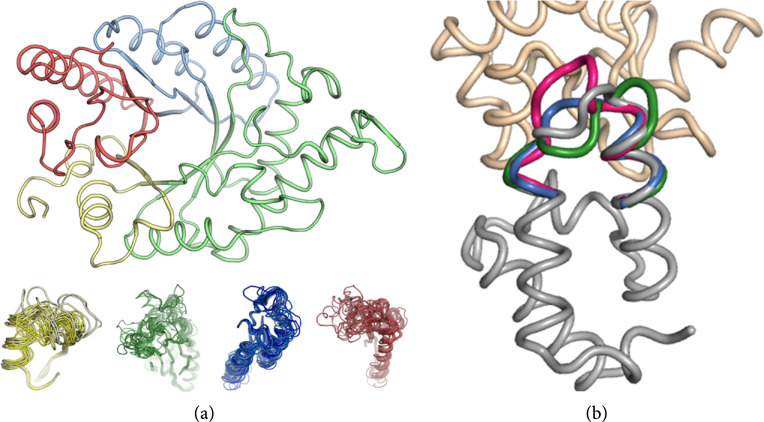
Modular assembly and design of large and functional proteins. (a) TIM barrel enzymes can be computationally segmented into modular backbone fragments that are then assembled and designed to generate new backbones. The bottom of the panel shows backbone fragments from homologs that can be modularly assembled instead of the same-colored fragments in the TIM barrel. (b) Backbone assembly and design of an interfacial loop (colors) in a binding pair of proteins (grey, PDB entry: 3u43) generate atomically accurate and highly specific binding pairs (PDB entries: 1e19 (green), 3q4q (blue), and 4rp4 (pink)).

To test the generality of modular assembly and design, we applied it to two additional long-standing challenges of protein design methodology. First, we generated new enzymes through fragment assembly and atomistic design (Figure 3(a)) [47]. Despite encoding more than 100 mutations from any natural enzyme, some of the designs were as stable and functional as natural enzymes in the same functional family, and some exhibited substantially different substrate selectivity profiles. Second, we used this strategy to recombine backbone fragments from nonhomologous proteins, generating new backbones and sequences in a high-affinity pair of interacting proteins [50]. This procedure yielded atomically accurate designs including in the new backbone and designed hydrogen-bonded networks (Figure 3(b)). Remarkably, some of the binding pairs exhibited very high binding specificity relative to the natural pair, demonstrating that accurate control over the backbone and sidechain degrees of freedom is the key to the design of high-specificity interactions. Taken together, these results suggested that evolution-guided atomistic design could provide a general solution to outstanding problems in protein design of function.

## 4. Reliable and Completely Computational Protein Optimization

Encouraged by finding that evolution-guided atomistic design could design accurate and stable new backbones, we turned to protein optimization. Here, we developed two complementary strategies: PROSS for optimizing protein native-state stability (by designing the sequence outside the active or binding site) [68] and FuncLib for designing stable and preorganized constellations of amino acid residues within enzyme active sites or protein binding sites [69]. In developing these design approaches, we relied on insights from decades of research on protein engineering and evolution [70] that demonstrated that (1) the vast majority of point mutations are neutral or deleterious to protein activity or stability [71]; (2) large differences in activity demand multipoint mutations in the active site, but multipoint mutants are even more likely than single-point mutations to destabilize or reduce protein activity levels [70]; and (3) the active-site constellation of amino acid residues is extremely sensitive even to remote mutations that may deform the protein backbone [72]. Additionally, we sought a general protein optimization framework that would only rely on data that are readily available, in principle, for any natural protein structure and not on protein-specific expertise.

The strategy that we developed uses data from multiple-sequence alignments of homologous proteins in addition to atomistic design calculations. In natural evolution, homologous proteins diverged from a common ancestor, and selection pressures ensured that all of the extant proteins retained their primary activity and foldability. Thus, a sequence alignment of natural homologs indicates which mutations are likely to be tolerated. Indeed, inferences from phylogenetic calculations have been successfully used in “consensus” design [73] and ancestral sequence reconstruction [74] for decades. They have also been implemented successfully in other atomistic stability design methods [36, 75]. Additionally, in order to mitigate the risk of deforming the active site, mutations are only accepted if they are predicted not to alter the catalytic constellation [50, 68, 69]. As a last step, we applied combinatorial sequence design either inside the active site to modify protein activity (in FuncLib [69]) or outside the active site to stabilize the protein (PROSS [68]).

For several decades, directed evolution has been the method of choice for protein optimization [76]. Although there are numerous examples of directed evolution successes, this method is iterative, laborious, and applicable only to systems that are amenable to medium or even high-throughput screening (>10^3^ and even >10^6^ variants). Many proteins, however, can only be assayed at low throughput, either because of complicated production requirements or because measuring their activity requires sensitive instruments. We started by applying PROSS and FuncLib to proteins that can only be assayed at low throughput, thus testing whether evolution-guided atomistic design is reliable enough to address “real-world” engineering challenges that are difficult or impossible for iterative methods. In these studies, we demonstrated that PROSS and FuncLib could dramatically improve protein expression levels, stability, and activity by testing a handful of designs (in the case of PROSS) [68, 77–79] or a few dozen (in the case of FuncLib) [50, 69, 80]. Due to their reliability, we enabled both methods as web servers. Opening these methods to general use had a profound impact on our understanding of the generality and reliability of these methods. In the following, we briefly describe lessons from studies mostly by other labs to address long-standing protein engineering problems using these methods.

## 5. Applied Protein Design

**Figure 4:**
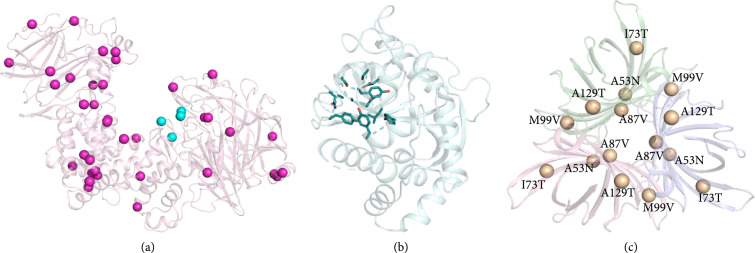
Representative applications of evolution-guided atomistic design. (a) Chondroitinase ABC was designed using the PROSS stability design method to improve its half-life in physiological conditions as a prerequisite for testing its use as a treatment for central nervous system injuries. 37 designed mutations indicated by purple spheres on the enzyme structure. Active site positions, at and around which no mutations were allowed, are indicated by cyan spheres (PDB entry: 2iip; the enzyme comprises >1,000 amino acids). (b) Active-site residues (sticks) in a nicotinamide N-methyltransferase (NMT, PDB entry: 1hn0) were designed using FuncLib to generate variants with high substrate regioselectivity for use in the production of alkylated pyrazoles for medicinal chemistry. (c) The homotrimer interfaces of PodA, a bacterial enzyme that degrades a critical metabolite in *P. aeruginosa* biofilms (PDB entry: 5k21), were designed to stabilize the assembly. The designs improved stability and expressibility enabling experiments to identify optimal conditions to eliminate these biofilms. Spheres indicate designed positions. PROSS and FuncLib are available online at https://PROSS.weizmann.ac.il and https://FuncLib.weizmann.ac.il.

In the first community benchmark of a design method, 12 labs applied PROSS to 14 diverse proteins [78]. Remarkably, in nine of these, at least one of the designs exhibited increased expression levels, and in 90% of the tested proteins, thermal stability improved in the designs. Furthermore, thermal stability increased more substantially in designs that incorporated a greater number of mutations. This observation suggested that the designed mutations were mostly additive and explained how the designs tolerate even large numbers of mutations (sometimes >50). Indeed, in a recent application, PROSS was successfully used to stabilize the bacterial chondroitinase ABC enzyme [81]. This enzyme comprises more than 1,000 amino acids and is, to our knowledge, the largest enzyme that has been successfully subjected to design calculations (Figure 4(a)). This enzyme is attractive due to its therapeutic potential to regenerate nerves in the aftermath of spinal cord injuries, but its low stability in the body (half‐life<1 day) has limited its applicability. By contrast, one of the PROSS designs exhibited prolonged stability (>4 days) and an increased level of activity.

The FuncLib method computes a set of active-site multipoint mutants for experimental testing [69]. Since substrate-bound and transition-state models are often inaccurate or difficult to compute, FuncLib design calculations can be applied to the enzyme *apo* state. In this case, the resulting designs do not target a specific substrate. Rather, the designs explore different active-site sequences, each of which is predicted to be stable and to stabilize the core catalytic amino acid residues in their functionally competent constellation. This strategy thus increases the likelihood that the designs would exhibit diverse selectivities and high activity. By changing active-site shape and electrostatics, FuncLib dramatically altered selectivity profiles generating nerve agent hydrolases that exhibited three orders of magnitude improved breakdown of toxic nerve agents compared to the natural enzyme which served as a starting point [69]. Furthermore, FuncLib was used to improve the regioselectivity of a nicotinamide N-methyltransferase (Figure 4(b)) [82]. In this study, nearly 30% of the designs exhibited improved activity (by up to two orders of magnitude) and one of the designs exhibited 99% regioselectivity. This design may be useful for the precise production of N-alkylated pyrazoles which are important intermediates in producing small-molecule therapeutics.

Enzyme active sites and protein-protein binding sites share in common a high density of amino acid interactions. We therefore also applied FuncLib to optimize protein-protein interactions, finding that it can improve protein-binding affinity [50] and antibody stability and affinity [83, 84] by optimizing atomic interactions across the interacting surfaces. Furthermore, improving the interactions across the homooligomeric interfaces in a trimeric bacterial enzyme called PodA (Figure 4(c)) led to improved stability and an order of magnitude increase in its production yields [85]. This enzyme is a candidate to serve as a novel antibiotic that targets recalcitrant *Pseudomonas aeruginosa* biofilms, and the design has enabled finding optimal treatment options.

As this brief summary shows, many challenges in basic and applied protein science are difficult to address with laboratory methods that demand high-throughput experiments. By contrast, the high reliability of evolution-guided atomistic design allows one-shot protein optimization through low-throughput experimental screening.

## 6. What Have We Learned from Evolution-Guided Atomistic Design?

We started exploring ways to incorporate evolutionary data in atomistic design calculations about a decade ago. At the time, we were frustrated with the incomplete control that atomistic design processes exerted over the design outcome [11, 20]: designed proteins exhibited low activity and often misfolded relative to the design conception. We assumed that by subjecting naturally occurring and versatile folds such as TIM barrels and antibodies to atomistic design, we would expose the design principles of natural and functional folds. We were particularly hopeful to identify negative-design principles—those principles that underlie the accurate folding of complex protein domains and rule out the myriads of misfolded (and nonfunctional) alternatives. In this section, we attempt to explain why evolution-guided atomistic design is reliable and what unexpected design principles we have learned from its application. We hope that these principles would be useful in protein design challenges that have not yet been addressed, not least, in the *de novo* design of large and functional proteins.

The failure of atomistic design calculations to reliably optimize stability and activity in large proteins was seen as a liability for many years [11, 86]. Speculations on the sources of error suggested that energy calculations are inherently inaccurate due to the approximate nature of the energy potentials related to solvation and electrostatics. Furthermore, the inability to provide a general framework to explain the mutational effects observed in protein engineering and directed-evolution experiments implied that perhaps protein optimization cannot be rationalized at all and would continue to rely on iterative experimental exploration [87]. By contrast with these views, however, evolution-guided protein stability design methods are able to improve the thermal stability and expressibility of probably more than half of the proteins subjected to them [78]. Some successfully designed proteins comprised hundreds and even more than 1,000 amino acids indicating that the design strategy is not very sensitive to the compounding of error in energy calculations which was held as a difficult challenge to overcome [11].

**Figure 5:**
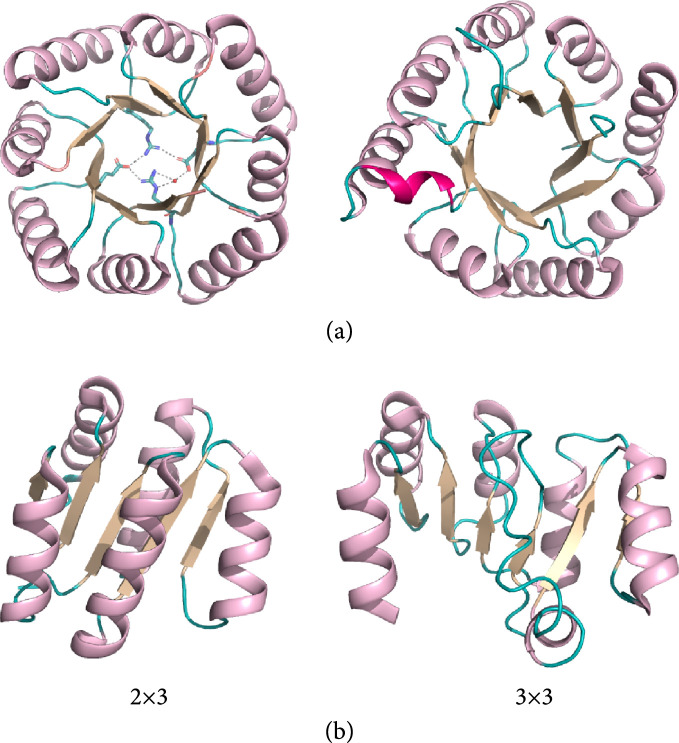
*De novo* designed proteins programmed to exhibit nonideal features. (a) TIM barrel structures, colored by secondary structure: helices (pink), beta-sheets (wheat), and loops (teal). (Left) A buried polar hydrogen bond network was designed to stabilize a *de novo* designed TIM barrel (PDB entry: 7p12, 88–90). (Right) A helix (magenta) was inserted in one of the loops to exhibit a more pocket-like structure reminiscent of natural active sites (PDB entry: 7a8s) [88–90]. (b) *De novo* designed ideal Rossmann fold structures. Large Rossmann fold structures (2×3 and 3×3, PDB entries: 6xeh and 7kbq, 113 and 129 amino acids, respectively) could be successfully designed only after adding strain in the form of *β*-sheet curvature [88–90].

Evolution-guided atomistic design owes its accuracy to eliminating mutations that are likely to destabilize the protein, induce misfolding, or distort the active-site constellation of residues [6]. Particularly, critical sequence and structure features that are not ideal, such as loops, buried polar interaction networks, and bent secondary structure elements, are maintained in evolution-guided atomistic design owing to the use of natural backbones and sequence constraints [44, 47–49]. Therefore, although atomistic design calculations, on their own, exhibit limited accuracy when applied to large proteins [49, 91], together with evolutionary constraints, they nonetheless exhibit high accuracy. The nonideal sequence and structural features are likely to serve a negative-design purpose as they drastically limit the number of nonnative low-energy conformations (Figure 2). Implementing such nonideal features may also provide an important key to increasing the size and fold complexity of *de novo* designed proteins (Figure 5) [88–90].

Dynamics is a critical determinant in many protein functions. The fact that protein design calculations are limited to considering one state (or a handful of states in some cases) severely restricts their ability to address dynamics. Furthermore, in some cases, dynamics and stability may trade-off, since stability design calculations introduce new stabilizing contacts to only one of the protein states [92]. Several of the proteins which were successfully designed using evolution-guided atomistic design processes nevertheless exhibit functionally important dynamics. Remarkably, the human estrogen receptor, which undergoes critical conformational changes in response to ligand binding, was subjected to PROSS stability design calculations, yielding a design with 24 mutations that improved its stability and yet maintained a very similar activation profile to the human protein [93]. Possibly, the dynamics that are critical to protein activity are maintained in these designs due to the sequence constraints derived from natural homologs. It is too early to say whether these intriguing results can be generalized to other dynamic proteins, and we are actively studying this question in other dynamic proteins.

Finally, the most important lesson has been to build on the insights gained over the past four decades of protein engineering and simulation. The critical importance of negative-design principles to counter misfolding and aggregation [8, 23, 94, 95], the reliability of sequence-based “consensus” design [73] and ancestral sequence reconstruction [74], the modularity of so many of the most versatile protein folds [96], and the fact that most mutations are neutral or deleterious [18, 71] have shaped our design strategy. We were also fortunate to have an intense and fruitful dialogue with one of the leaders of modern enzyme evolution and engineering, Dan Tawfik, who tragically died last year [97]. He made profound contributions to clarifying these principles [98–101] and insisted that design methods should be tested on real-world protein engineering challenges, collaborating with us to design large proteins that were intransigent even to the most reliable computational and experimental optimization strategies [68, 69, 80].

## 7. Outlook

The most dramatic development in computational structural biology of the recent decade is the emergence of deep learning-based ab initio structure predictors such as AlphaFold2 [102] and RoseTTAFold [103] that generate atomically accurate model structures directly from sequence. Using these methods, essentially any protein can be accurately modeled without requiring large computational resources. This is an exciting development for structure-based protein optimization methods since they can now be used to generate functionally expressed designs even in proteins that are so unstable that they had not previously been characterized experimentally [104]. This combined modeling and design strategy, therefore, goes beyond mere optimization of known activities to discover new activities encoded in natural proteins. We believe that this combined strategy will contribute significantly to research and utilization of proteins that are critical to human health, industry, and the environment but have not yielded to experimental characterization.

Looking beyond the optimization of natural proteins, a long-standing goal of protein design methodology is to design new activities completely from scratch. It is still unclear, however, how to use evolutionary data in guiding the design of activities that are not encoded in nature [105]. Recent results using deep learning-based predictors suggest an intriguing possibility that they may be able to assess the foldability of protein designs [106–108]. These methods may therefore replace evolutionary data in ensuring that designs accurately fold as conceived. Thus, the next phase of innovation in protein design methodology is likely to rely in part on statistical learning methods. These may open the way to one of the most long-standing goals of protein engineering: the completely computational design of new or improved molecular activities without recourse to experimental data.
